# Minocycline Attenuates Cognitive Impairment Induced by Isoflurane Anesthesia in Aged Rats

**DOI:** 10.1371/journal.pone.0061385

**Published:** 2013-04-17

**Authors:** Feijuan Kong, Shuping Chen, Yuan Cheng, Leilei Ma, Huishun Lu, Honghai Zhang, Wenwen Hu

**Affiliations:** 1 Department of Anesthesiology, Hangzhou First People's Hospital, Nanjing Medical University, Hangzhou, China; 2 Department of Anesthesiology, Second Affiliated Hospital, School of Medicine, Zhejiang University, Hangzhou, China; 3 Department of Anesthesiology, Women's Hospital, School of Medicine, Zhejiang University, Hangzhou, China; University of Victoria, Canada

## Abstract

Postoperative cognitive dysfunction (POCD) is a clinical phenomenon characterized by cognitive deficits in patients after anesthesia and surgery, especially in geriatric surgical patients. Although it has been documented that isoflurane exposure impaired cognitive function in several aged animal models, there are few clinical interventions and treatments available to prevent this disorder. Minocycline has been well established to exert neuroprotective effects in various experimental animal models and neurodegenerative diseases. Therefore, we hypothesized that pretreatment with minocycline attenuates isoflurane-induced cognitive decline in aged rats. In the present study, twenty-month-old rats were administered minocycline or an equal volume of saline by intraperitoneal injection 12 h before exposure to isoflurane. Then the rats were exposed to 1.3% isoflurane for 4 h. Two weeks later, spatial learning and memory of the rats were examined using the Morris Water Maze. We found that pretreatment with minocycline mitigated isoflurane-induced cognitive deficits and suppressed the isoflurane-induced excessive release of IL-1β and caspase-3 in the hippocampal CA1 region at 4 h after isoflurane exposure, as well as the number of TUNEL-positive nuclei. In addition, minocycline treatment also prevented the changes of synaptic ultrastructure in the hippocampal CA1 region induced by isoflurane. In conclusion, pretreatment with minocycline attenuated isoflurane-induced cognitive impairment in aged rats.

## Introduction

Postoperative cognitive dysfunction (POCD), a major clinical issue, is described as cognitive deficits in memory and concentration after anesthesia and surgery, especially in geriatric surgical patients. Studies have shown that about 25% of elderly (60 years or older) patients exhibit POCD 1 week after non-cardiac surgery, and about 10% of elderly patients exhibit POCD 3 months after non-cardiac surgery [1], [2]. POCD may be self-limiting in most patients [3]–[5], but in some patients, it is long-term or even permanent [6]. In addition to affecting patients’ prognosis and quality of life, POCD has been shown to be associated with an increased incidence of postoperative complications and mortality [7], [8].

With the acceleration of the aging population and the development of medical technology, the opportunity of the elderly for surgery has increased significantly in recent years. Most patients have their surgery performed under general anesthesia [9]. Inhalation anesthetics such as isoflurane have been widely used in recent years in clinical and research practices. Although the current clinical data does not provide a strong link between anesthesia and cognitive impairment, a number of preclinical studies demonstrate that exposure to volatile anesthetics causes cognitive impairment for days or weeks in aged animals [10]–[14]. These observations raise concerns about the potentially deleterious effects of general anesthesia in elderly patients.

The pathogenesis of volatile anesthetic-induced cognitive impairment is not fully understood. During the past few years, an increasing amount of evidence has supported the view that the excessive release of proinflammatory cytokines, including tumor necrosis factor (TNF)-α, interleukin (IL)-1β and IL-6, is involved in cognitive impairment after surgery and anesthesia [14]–[16]. A recent study suggested that volatile anesthetic isoflurane increases the levels of TNF-α, IL-6, and IL-1β in brain tissues and primary neurons of mice [16]. The cytokines are released from several cell types, and can be synthesized in the central nervous system by microglia, astrocytes, and some populations of neurons. IL-1β increases the expression of nearly all other cytokines such as TNF-α, IL-6, and chemokines as well as adhesionmolecules. TNF-α and IL-1β are bone arrow stimulants that increase the number of myeloid progenitor cells and promote the release of neutrophils, resulting in neutrophilia to the site of inflammation and enhanced blood–brain barrier permeability [15]. In addition, hippocampal neuronal apoptosis was postulated to be associated with the cognitive dysfunction caused by isoflurane [14], [17]. Our recent study also demonstrated isoflurane exposure increased activated caspase-3, a key enzyme involved in cell apoptosis in rat hippocampus, as well as CHOP and caspase-12, key mediators of endoplasmic reticulum stress-mediated cell death in the hippocampus [18], [19].

POCD has drawn significant attention from the public and scientific community, however, there are few clinical interventions and treatments available to prevent this disorder. Minocycline, a tetracycline derivative, has been reported to have neuroprotective effects [20], [21]. The aims of this study were to detect whether pretreatment with minocycline attenuates isoflurane-induced cognitive decline in aged rats.

## Materials and Methods

### Animals

All of the animals were treated according to the guidelines of the Guide for the Care and Use of Laboratory Animals (United States National Institutes of Health). The Laboratory Animal Care Committee of Zhejiang University approved all experimental procedures and protocols. All efforts were made to minimize the number of animals used and their suffering. The rats were housed in polypropylene cages, and the room temperature was maintained at 22°C, with a 12-hour light–dark cycle. Twenty-month-old male Sprague-Dawley rats, weighing 350–400 g, were used for all experiments.

### Exposure to Anesthetic

The rats were randomly divided into for groups: control, minocycline, isoflurane and minocycline-isoflurane (n = 20 in each group). Rats in the minocycline and minocycline-isoflurane groups received minocycline (45 mg/kg) by intraperitoneal injection 12 h before exposure to isoflurane. Rats in the other two groups were intraperitoneally injected with an equal volume of saline. The minocycline dose used in our study (45 mg/kg, i.p.) was chosen based on the studies by others [20], [22]. All the rats were placed in plastic containers resting in water baths with a constant temperature of 38°C. In these boxes, rats in the isoflurane and minocycline-isoflurane groups were exposed to 1.3% isoflurane (Lot 826005U, Abbott Laboratories Limited, USA) in a humidified 30% oxygen carrier gas for 4 h; the control and minocycline groups were exposed to simply humidified 30% oxygen without any inhalational anesthetic for 4 h. The 1.3% concentration was chosen because it represents one minimum alveolar concentration (MAC, the concentration at which 50% of animals do not have a motor response to painful stimuli) of isoflurane in rats. General anesthesia maintained with anesthetics including volatile anesthetics at 0.5∼1.3 MAC for 2 h or longer is commonly performed in clinical practice. The determination of anesthetic duration was based on our preliminary study, which indicated that physiological states of the rats remained stable throughout a 4-hour isoflurane exposure. The isoflurane concentration, oxygen and carbon dioxide levels in the box were monitored with an agent gas monitor (Vamos, Drager Medical AG & Co. KgaA, Germany). Otherwise, animals in all groups were under the same treatment and environment. The rectal temperature was maintained at 37 ± 0.5°C. Mean blood pressure (MAP) and heart rate were measured before and during anesthesia with a noninvasive blood pressure meter (Kent Scientific Corp., Torrington, CT, USA). Arterial blood gases (ABG) and blood glucose were measured at the end of the 4-hour anesthetic exposure.

At 4 h after exposure, five rats in each group were sacrificed, and the hippocampi were dissected for detecting the levels of IL-1β, TNF-α and IL-6 by ELISA and neuronal apoptosis.

Two weeks after anesthesia exposure, the animals were subjected to a Morris Water Maze test to determine their cognitive function. Following the Morris Water Maze test, the rats were sacrificed, and the hippocampi were removed for evaluation of IL-1β, TNF-α and IL-6 by ELISA, caspase-3 by Western blot, TUNEL staining and the ultrastructure changes of synapses by transmission electron microscopy (TEM).

### Memory and Learning Studies

The Morris Water Maze test was performed as previously described [23]. A round pool (diameter, 150 cm; depth, 50 cm) was filled with warm (24°C) opaque water to a height of 1.5 cm above the top of the movable clear 15-cm-diameter platform in the third quadrant. A video tracking system recorded the swimming motions of animals, and the data were analyzed using motion-detection software for the Morris Water Maze (Actimetrics Software, Evanston, IL, USA). After every trial, each rat was wiped dry and kept warm before returning to its regular cage, where it had free access to food.

The place trials were performed on the 15th day after isoflurane exposure for four days to determine the rats’ ability to obtain spatial information. A dark black curtain surrounded the pool to prevent confounding visual cues. All rats received 4 trials per day in each of the four quadrants of the swimming pool. On each trial, rats were placed in a fixed position into the swimming pool facing the wall. They were allotted 120 sec to find the platform in the third quadrant upon which they sat for 20 sec before being removed from the pool. If a rat did not find the platform within 120 sec, the rat was gently guided to the platform and allowed to remain there for 20 sec. For all training trials, swim speed and the time to reach the platform (escape latency) were recorded. The less time it took a rat to reach the platform, the better the learning ability. We took the average of four trials as the escape latency each day.

Probe trials were conducted immediately after the four-day period to evaluate memory retention capabilities. The probe trials involved removing the submerged platform in the third quadrant from the pool and allowing the rats to swim for 120 sec in any of the four quadrants of the swimming pool. The number of original platform crossings and time spent in the third quadrant were recorded.

### Transmission Electron Microscopy

The animals were anesthetized with a lethal dose of Nembutal. The thoracic cavities were opened and perfused intracardially with 100 mL of normal saline. Then the hippocampus, including CA1 area, of each rat was taken out immediately. Immersion fixation was completed on tissues about 1 mm^3^ from the hippocampus. Samples were rinsed in cold phosphate-buffered saline (PBS) and placed in 2.5% glutaraldehyde at 4°C for 4 h. The tissue was rinsed in buffer and post-fixed with 1% osmium tetroxide for 1 h. Then, the tissue was rinsed with distilled water before undergoing a graded ethanol dehydration series and was infiltrated using a mixture of half propylene oxide and half resin overnight. Twenty-four hours later, the tissue was embedded in resin. 120 nm sections were cut and stained with 4% uranyl acetate for 20 min and 0.5% lead citrate for 5 min. Ultrastructural changes of synapses in the hippocampi were observed under a transmission electron microscope (Philips Tecnai 10, Holland). Electron microscope photographs were analyzed using Image-Pro Plus 6.0 software (Media Cybernetics, Silver Spring, MD, USA). The area of the postsynaptic density (PSD) and the synaptic cleft were calculated as described previously [24].

### Western Blot Analysis

The rats were anesthetized with a lethal dose of Nembutal. Their thoracic cavities were opened and perfused intracardially with 100 mL of normal saline. The hippocampus, including CA1 and dentate gyrus field, of each rat was taken out immediately to obtain fresh tissue specimens. Hippocampal tissues were homogenized on ice with 2 mM phenylmethanesulfonyl fluoride in 1 mL ice-cold RIPA buffer added protease inhibitor cocktail EDTA-free. Homogenates were centrifuged at 13,000×g at 4°C for 30 min. The supernatant was saved and its protein concentration was determined by the BCA method using bovine serum albumin as the standard. Protein samples (50 µg) were separated by 12% sodium dodecyl sulfate polyacrylamide gel electrophoresis (SDS-PAGE) and transferred to a nitrocellulose membrane (Wuhan Boster Biological Technology., Ltd, China). The membranes were blocked by nonfat dry milk buffer for 2 hours and then incubated overnight at 4°C with primary antibody against caspase-3 (1∶1000, Santa Cruz Biotechnology, USA). The membranes were subsequently incubated with HRP-conjugated secondary antibodies and detected with enhanced chemiluminescence (ECL) detection reagent (Amersham Biosciences, Piscataway, NJ). The optical densities of bands were quantitatively analyzed using Bio-Rad Quantity One 4.6.2 (Bio-Rad Laboratories, USA). The results were expressed as a relative density. Equal protein loading in each lane was confirmed by hybridization with a 1∶2000 dilution of β-actin antibody (Santa Cruz Biotechnology, USA).

### TUNEL Staining

The rats were anesthetized with a lethal dose of Nembutal. The aorta was cannulated and the animal was firstly perfused with 200 mL of normal saline, then with 250 mL of 4% formaldehyde (freshly made from paraformaldehyde) for 20–30 min. The fixed brain was then removed from the cranial cavity and post-fixed overnight in the same fixative at 4°C. The tissues were embedded in paraffin, and transverse paraffin sections containing the hippocampi were mounted on silanecoated slides. Sections were deparaffinaged and rehydrated. Then the sections were treated for antigen retrieval with 10.2 mmol/L sodium citrate buffer, pH 6.1, for 20 min at 95°C for TUNEL staining. The brain tissue sections were stained using an in situ cell death detection kit (POD; Roche Diagnostics Corp., Indianapolis, IN, USA), following the manufacturer’s protocol. Ten microscopic fields (400 ×) from each section were assayed by counting brown nuclei. The percentage of TUNEL-positive nuclei (brown nuclei) in the hippocampal CA1 region was calculated.

### Enzyme-Linked ImmunoSorbent Assay (ELISA)

The rats were anesthetized with a lethal dose of Nembutal. Their thoracic cavities were opened and perfused intracardially with 100 mL of normal saline. The hippocampus, including CA1 field, of each rat was taken out immediately. Hippocampal tissues were homogenized on ice in 20 mM Tris-HCl buffer (pH = 7.3) containing protease inhibitors (10 mg/ml aproteinin, 5 mg/ml peptastin, 5 mg/ml leupeptin, and 1 Mm phenylmethanesulfonylfluoride). Homogenates were centrifuged at 10,000×g for 10 min at 4°C. The supernatant was then ultracentrifuged at 150,000×g for 2 h. Bradford protein assay of the supernatant was performed for each sample. The protein levels of IL-1β, TNF-α and IL-6 in the hippocampal tissues were determined by commercially available ELISA kits (Santa Cruz Biotechnology, USA) following the protocols provided by manufacturer. All samples were assayed in duplicate. The readings were normalized to the amount of standard protein.

### Statistical Analysis

All data were presented as mean ± S.E.M. Results of values of MAP, heart rate, ABG and place trials of aged rats were analyzed using 2-way ANOVA for repeated measurements. Other data were analyzed using one-way ANOVA, followed by Tukey post hoc multiple comparison tests. A *P* value of <0.05 was considered statistically significant. All statistical tests and graphs were performed or generated, using GraphPad Prism Version 4.0 (GraphPad Prism Software, Inc. CA, USA).

## Results

### Physiologic Parameters

**Figure 1:**
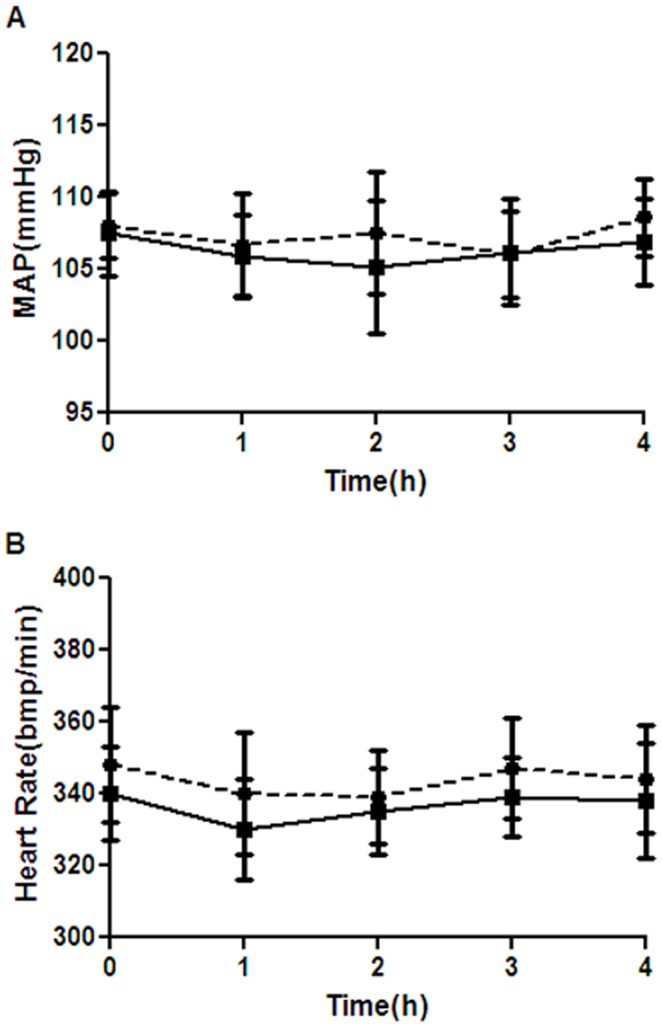
Isoflurane anesthesia had no significant effects on MAP (A) and heart rate (B). *Solid circle* = control group; *solid square* = isoflurane treated groups included isoflurane and minocycline-isoflurane groups. Data represent mean ± S.E.M. n = 40 for each time point.

As shown in Fig. 1, MAP (Fig. 1 A) and heart rate (Fig. 1 B) decreased after isoflurane exposure, but the difference was not statistically significant. As shown in Table 1, ABG values and blood glucose levels were within the normal physiologic range. There were no significant differences among the four groups on any measured variables for ABG values and blood glucose levels. This data rules out the possibility that isoflurane-induced neurodegeneration in the brains was caused by physiologic side effects (e.g. hypotension, hypoglycemia, hypoxia and hypercapnia).

**Table 1:** Physiological parameters during isoflurane exposure.

	Con	Mino	Iso	Mino-Iso
pH	7.36 ± 0.04	7.39 ± 0.05	7.37 ± 0.04	7.41 ± 0.06
PaCO_2_ (mmHg)	35.4 ± 3.2	36.8 ± 2.6	38.7 ± 3.6	37.6 ± 2.3
PaO_2_ (mmHg)	168 ± 14.6	159 ± 12.5	160 ± 16.8	164 ± 15.5
SaO_2_ (%)	98.7 ± 1.2	97.3 ± 1.3	98.1 ± 1.8	97.4 ± 1.6
Glucose (mg/dl)	116 ± 14	117 ± 16	115 ± 17	118 ± 21

Isoflurane exposure did not affect arterial blood gas values and blood glucose levels significantly. Values are mean ± S.E.M. n = 5 for each group.

Con = control; Mino = minocycline; Iso = isoflurane; PaCO_2_ = arterial carbon dioxide tension; PaO_2_ = arterial oxygen tension; SaO_2_ = arterial oxygen saturation.

### Minocycline Attenuated Isoflurane-induced Cognitive Impairment

**Figure 2:**
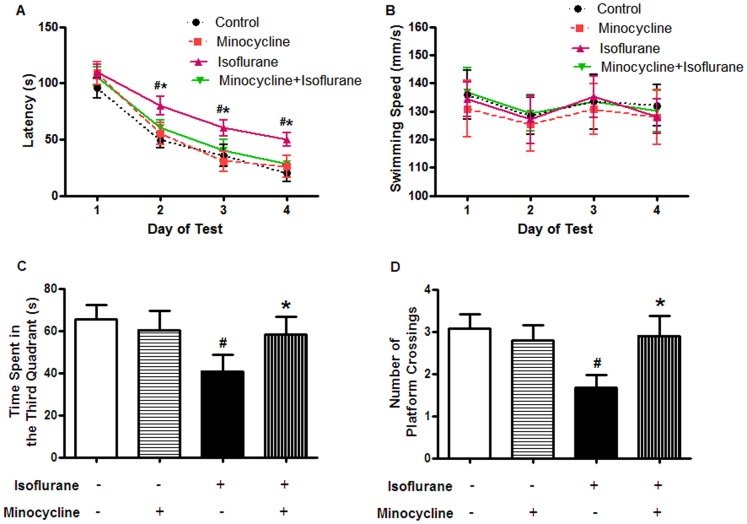
Minocycline pretreatment attenuated isoflurane-induced cognitive impairment in the Morris Water Maze test. (A, B) Place trial demonstrating the latency for the rats to reach platform (A) and the swimming speed (B) measuring spatial information acquisition. (C, D) Probe trial demonstrating the number of original platform crossings (C) and the time spent in the third quadrant (D) measuring memory retention capabilities. Data represent mean ± S.E.M. n = 15 in each group.^ #^ *P*<0.05 isoflurane group compared with control group; *P<0.05 minocycline-isoflurane group compared with isoflurane group.

As shown in Fig. 2 A, rats in all groups showed a rapid decrease in latency. In the place trial, the rats in the minocycline-isoflurane group spent less time to find the platform than those in the isoflurane group (*P*<0.05). There was no significant difference in the latency between the minocycline-isoflurane and control groups. Swimming speeds were also analyzed during place trials, and no differences were observed among the four groups (Fig. 2 B, *P*>0.05).

In the probe test, the rats in the minocycline-isoflurane group spent more time in the third quadrant where the platform was located compared with those in the isoflurane group (Fig. 2 C, *P*<0.05). There was an increase in the number of crossings over the former platform location (Fig. 2 D, *P*<0.05). There was no significant difference between the minocycline-isoflurane and control groups.

### Minocycline Decreased the Levels of IL-β and Caspase-3 in the Hippocampus after Isoflurane Anesthesia

**Figure 3:**
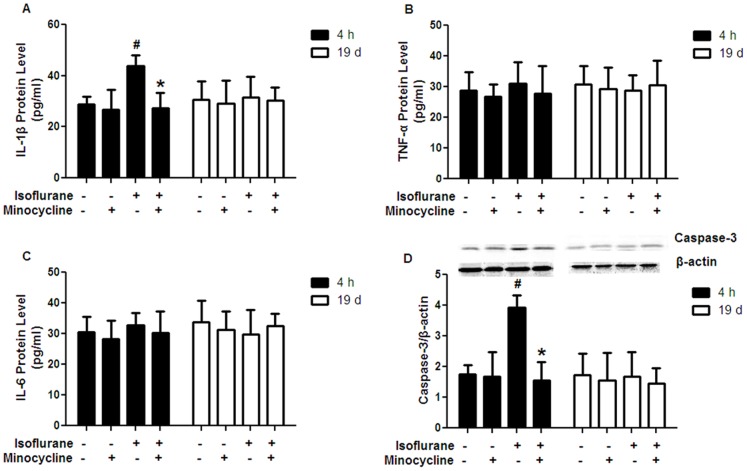
Effects of minocycline pretreatment on the levels of IL-1β, TNF-α, IL-6 and caspase-3 in the hippocampus. (A) Minocycline application decreased the level of IL-1β at 4 h after isoflurane exposure, but not at 19 d after isoflurane exposure. (B) No differences were observed in TNF-α level among the four groups at 4 h or 19 d after isoflurane exposure. (C) No differences were observed in IL-6 level among the four groups at 4 h or 19 d after isoflurane exposure. (D) Minocycline application decreased the protein level of caspase-3 at 4 h after isoflurane exposure, but not at 19 d after isoflurane exposure. A representative Western blot is shown and the quantified caspase-3 bands were normalized to the loading control β-actin. 4 h = 4 h after isoflurane exposure; 19 d = 19 days after isoflurane exposure. Data represent mean ± S.E.M. n = 5 in each group.^ #^ *P*<0.05 isoflurane group compared with control group; *P<0.05 minocycline-isoflurane group compared with isoflurane group.

Isoflurane exposure dramatically increased the levels of IL-1β and caspase-3 in the hippocampal CA1 region at 4 h after isoflurane exposure compared with the control. This increase was attenuated by minocycline application (Fig. 3 A and Fig. 3 D, *P*<0.05). There was no significant difference between the minocycline-isoflurane and control groups. However, the levels of TNF-α and IL-6 in the hippocampal CA1 region was not different among the four groups at 4 h after isoflurane exposure (Fig. 3 B and C).

In contrast to the findings during the acute phase following isoflurane exposure, there were no differences among the four groups with regard to the IL-β, TNF-α or IL-6 concentrations as well as the caspase-3 expression in the hippocampal CA1 region at 19 d after isoflurane exposure (Fig. 3).

### Minocycline Inhibited Isoflurane-induced Apoptosis in the Hippocampus

**Figure 4:**
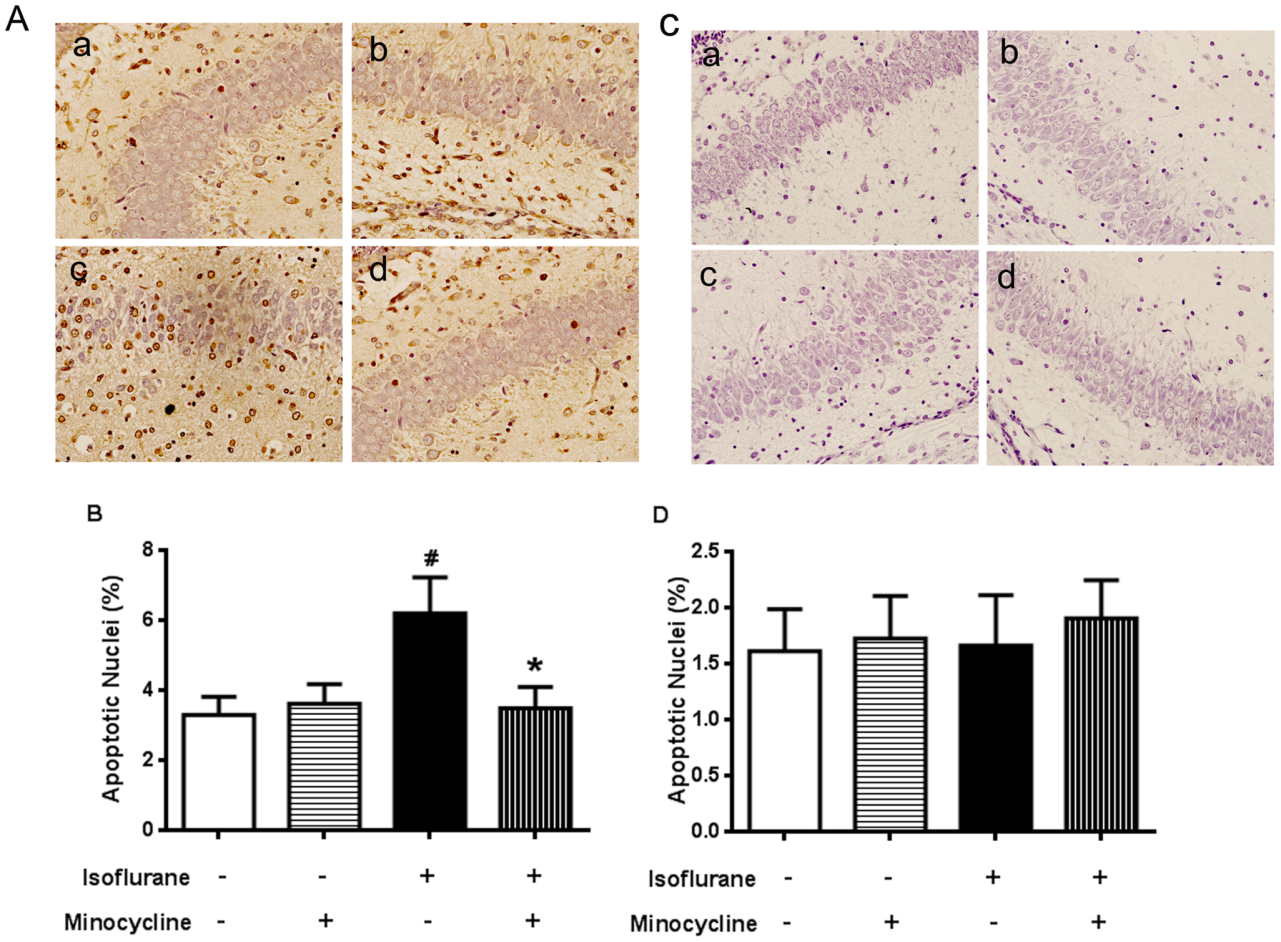
Effects of minocycline pretreatment on neuronal apoptosis in the hippocampal CA1 region. (A, C) TUNEL staining at 4 h (A) and 19 d (C) after isoflurane exposure. (A a, C a) Control group (A b, C b) Minocycline group (A c, C c) Isoflurane group (A d, C d) Minocycline-Isoflurane group. Quantitation of the data is represented by the graph in panel B and D. Data represent mean ± S.E.M. n = 5 in each group.^ #^ *P*<0.05 isoflurane group compared with control group; *P<0.05 minocycline-isoflurane group compared with isoflurane group.

As shown in Fig. 4, the number of TUNEL-positive nuclei expressed as a percentage of total nuclei was markedly increased in the isoflurane group over the control group in the hippocampal CA1 region at 4 h after isoflurane exposure. The increase was inhibited by minocycline application (Fig. 4 A and Fig. 4 B, *P*<0.05). There was no significant difference between the minocycline-isoflurane and control groups.

However, the number of TUNEL-positive nuclei in the hippocampal CA1 region was not different among the four groups at 19 d after isoflurane exposure (Fig. 4 C and D).

### Minocycline Mitigated Isoflurane-induced Impairment in the Synaptic Ultrastructure of Hippocampus

**Figure 5:**
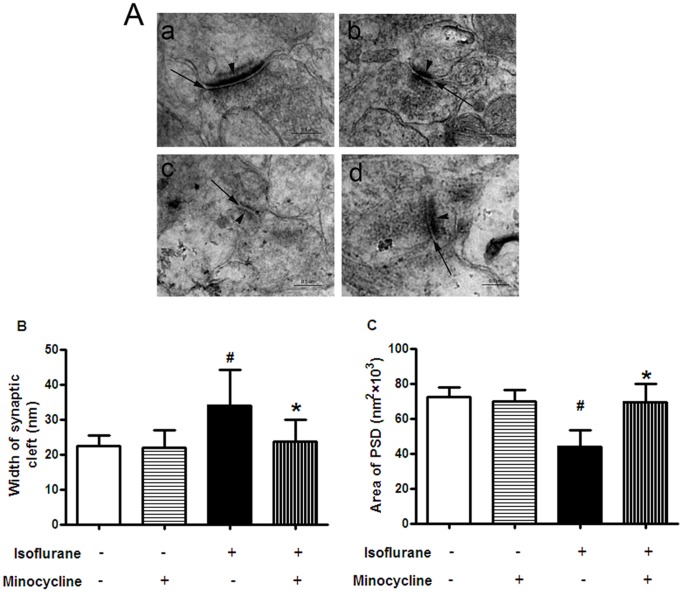
Minocycline alleviated impairment in the synaptic ultrastructure of the hippocampal CA1 region after isoflurane anesthesia. (A) Ultrastructural changes of synapses in the hippocampal CA1 region under TEM. (A a) Control group (A b) Minocycline group (A c) Isoflurane group (A d) Minocycline-Isoflurane group. Arrows = synaptic cleft; arrowheads = postsynaptic densities. Scale bar = 0.5 µm. Quantitation of the data is represented by the graph in panel B (Width of synaptic cleft) and C (Area of PSD). Data represent mean ± S.E.M. n = 5 in each group.^ #^ *P*<0.05 isoflurane group compared with control group; *P<0.05 minocycline-isoflurane group compared with isoflurane group.

To investigate the mechanism of cognitive impairment induced by isoflurane anesthesia, the synaptic morphometry changes in the hippocampal CA1 region were observed with TEM. Compared with control group, the synaptic cleft widened and the area of postsynaptic densities (PSD) remarkably decreased in isoflurane group (Fig. 5 A c, Fig. 5 B and C, *P*<0.05). These impairments were attenuated by minocycline application (Fig. 5). There was no significant difference between the minocycline-isoflurane and control groups.

## Discussion

The major finding of the present study was that pretreatment with minocycline attenuated cognitive decline induced by a clinically relevant concentration of isoflurane in aged rats. In addition, minocycline treatment also suppressed the excessive release of IL-1β and neuronal apoptosis and prevented the impairment of synaptic ultrastructure in the hippocampal CA1 region induced by isoflurane.

Learning and memory are important aspects of cognitive function. To determine the effect of minocycline on cognitive function after isoflurane anesthesia, the Morris Water Maze was used to assess learning and memory in aged rats. The Water Maze protocol evaluates learning and memory that involves a sequence of specific molecular processes in the CA1 area of the hippocampus [25]. The place trials were performed to determine the rats’ ability to obtain spatial information and the probe trials were conducted to evaluate memory retention capabilities. Twenty-month-old rats were used for all experiments in this study, because rats at this age may be considered as in their early elderly stage or late phase of middle age. Cognitive function tests on the animals were initiated at 2 weeks after isoflurane exposure, because most patients have already been discharged from hospital by this time. In the current study, our results showed that exposure of these rats to 1.3% isoflurane for 4 h caused deficits in the spatial learning and memory as manifested by the longer escape latency to reach the platform, the fewer times of original platform crossing and the less time spent in the target quadrant in the Morris Water Maze test. The lack of differences in swimming speeds of all groups excluded the possibility that sensorimotor disturbances in any of the groups could have influenced the learning and memory changes observed in our study. Since the Morris Water Maze test is considered to be hippocampus-dependent [25], our results suggest that isoflurane impaired hippocampus-dependent learning and memory in rats. These results are consistent with our previous studies [18], [19] and other related reports [10]–[14]. However, the effects of anesthesia on memory and learning are controversial, with transient improvement [26], no effects [27] and permanent impairment [10]–[14], [18], [19] all being reported. Differences in methods of anesthetic exposure, animal species (rats vs. mice), pharmacology (isoflurane vs. sevoflurane), anesthetic concentrations (0.5∼2 MAC), anesthetic durations (1–6 h), time of isoflurane exposure, and time to perform the leaning and memory tests may have contributed to these discrepancies.

Minocycline is a semi-synthetic second-generation tetracycline, which is a highly lipophilic molecule that easily crosses the blood–brain barrier (BBB) [28]. In addition to its own antimicrobial activities, minocycline has been reported to exert neuroprotective effects over various experimental models such as cerebral ischemia [29], Spinal Cord Injury [22], Parkinson’s disease (PD) [30], HD [21], and AD [20]. In the present study, we showed that pretreatment with minocycline attenuated isoflurane-induced learning and memory impairments. However, the efficacy of minocycline varies from robust protection [20]–[22], [29], [30], to no effect [31], to exacerbation of impairment [32]. This variability may arise from different dosage regimens, animal models and methodological differences. In animals, minocycline is lethal at very high doses [33], [34]. In humans, long-term treatment with minocycline at doses of up to 200 mg/day is generally safe and well tolerated as demonstrated by tolerability tests and clinical trials in rheumatoid arthritis, acne vulgaris, and HD [33], [35]. The dose of minocycline used in the present study was chosen based on the studies by others [20], [22].

The mechanisms of inhalation anesthetic-mediated neurodegeneration are still not clear. Minocycline has anti-inflammatory properties that are completely separate and distinct from its antimicrobial actions. Since minocycline attenuated isoflurane-induced cognitive impairment, it is possible that isoflurane induces neuroinflammation then leads to cognitive dysfunction. Consistent with this idea, it has been shown that cognitive impairment is associated with neuroinflammation in different brain regions, including the hippocampus [36], [37]. In addition, several studies have found that volatile anesthetics might cause neuroinflammation [14], [16]. A recent study showed that tibial surgery under general anesthesia triggered an IL-1β-mediated inflammatory process in the hippocampus that underlies memory impairment in young adult mice [16]. In agreement, our study showed that isoflurane exposure dramatically increased the level of IL-1β in the hippocampal CA1 region at 4 h after isoflurane exposure and this excessive release was reversed by minocycline pretreatment. Moreover, we measured proinflammatory cytokines IL-1β, IL-6 and TNF-a concentration at a time when animals had significant cognitive impairments. Our results showed that isoflurane induced learning and memory impairment, however, did not increase the level of IL-1β, IL-6 or TNF-a. Thus, our results did not indicate a role of neuroinflammation in isoflurane-induced cognitive dysfunction in the elderly rats.

It has been proposed that brain cell death after anesthetic exposure may contribute to the brain functional changes [18], [19], [38]. Isoflurane has been demonstrated to increase activated caspase-3 in vivo and vitro models [19], [20]. This activation may result in cell apoptosis and brain structure changes [19]. In our study, isoflurane exposure increased caspase-3 expression and the number of TUNEL-positive nuclei in the hippocampal CA1 region at 4 h after the exposure and minocycline attenuated this increase. In consideration of ultrastructural changes of synapses, our results suggest that the possible cell injury after isoflurane exposure may contribute to neurodegeneration and consequent deficits in learning and memory.

Synaptic transmission is essential for nervous system function, and its dysfunction is a known major contributing factor to cognitive impairment [39]–[41]. The synaptic cleft is a region of information transmission among neurons and plays an important role in the dynamics of synaptic activity. The postsynaptic density (PSD) is the material basis of synaptic efficacy. The area of PSD and the ability of learning and memory training and memory retention go hand in hand [40], [42]. In the present study, we found that the synaptic cleft was widened and the area of PSD was visibly reduced by isoflurane anesthesia, and these changes were attenuated by minocycline. Consistent with our result, long-lasting disturbances in the ultrastructural properties of developing synapses caused by inhalational anesthetics have been demonstrated in young rats [43]. In addition, inhalational anesthetics have been shown to disrupt postsynaptic density 95, discs large, and zonula occludens-1 domain-mediated protein–protein interactions, which provide a framework for the assembly of multiprotein signaling complexes at PSD [44], [45]. Our recent studies have found that learning and memory impairments were accompanied by the changes of normal structure of synapses, including a widened synaptic cleft and a decreased area of PSD [18], [19], [46]. Taken with previous studies, our results suggest that cell injury-mediated impairments in synaptic structure and function may be responsible for cognitive deficits induced by isoflurane in aged rats, and minocycline may prevent or reverse these changes.

Previous studies show that hypotension, hypoglycemia, hypoxia, hypercapnia and unstable body temperature during isoflurane anesthesia may impair cognitive function [1], [47]. To exclude these side effects, mean blood pressure, heart rate and body temperature were dynamically monitored during anesthetic exposure. ABG analysis and blood glucose were measured at the end of isoflurane exposure. No significant changes were found in any of these parameters. In the present study, no animals died after 4 h of isoflurane exposure, which indicated that even if some minimum physiological changes occurred, they were of little clinical relevance.

In conclusion, our study showed that exposure to a clinically relevant concentration of isoflurane induced learning and memory impairment in old rats. This detrimental effect was attenuated by minocycline pretreatment before isoflurane exposure.
